# Predictors of Early and Late Mortality for Patients with Hematologic Malignancy and Invasive Mold Disease

**DOI:** 10.3390/jof7090697

**Published:** 2021-08-27

**Authors:** Eva L. Yashphe, Ron Ram, Irit Avivi, Ronen Ben-Ami

**Affiliations:** 1 Internal Medicine T, Tel Aviv Sourasky Medical Center, Tel Aviv 6423906, Israel; jambeva@gmail.com; 2 Department of Hematology and Stem Cell Transplantation Service, Tel Aviv Sourasky Medical Center, Tel Aviv 6423906, Israel; ronr@tlvmc.gov.il (R.R.); iritavi@tlvmc.gov.il (I.A.); 3 Sackler Faculty of Medicine, Tel Aviv University, Tel Aviv 6997801, Israel; 4 Infectious Diseases Department, Tel Aviv Sourasky Medical Center, Tel Aviv 6423906, Israel

**Keywords:** invasive mold infection, survival analysis, hematologic malignancy, stem cell transplantation

## Abstract

Background: Invasive mold infections (IMI) are leading infectious causes of mortality among patients with hematological malignancies. Objectives: To determine the relative contribution of host, disease, and treatment-related factors to patient survival. Methods: An observational, retrospective cohort study reviewing the medical records of patients with hematological malignancy and IMI (2006–2016). Causes of death were classified up to 90 days after diagnosis. Kaplan–Meier and Cox regression analyses were used to determine risk factors for early, late, and overall mortality. Results: Eighty-six patients with IMI were included; 29 (34%) and 41 (47%) died within 6 and 12 weeks of diagnosis, respectively. Death was attributed to IMI in 22 (53.6%) patients, all of whom died within 45 days of diagnosis. Risk factors for early mortality were elevated serum galactomannan, treatment with amphotericin B, IMI progression 3 weeks after diagnosis, and lymphoma undergoing HCT. Late mortality was associated with relapsed/refractory malignancy and elevated serum galactomannan. Conclusions: In this single-center study of patients with IMI, infections were the most frequent causes of death, and time-dependent risk factors for death were identified. These results may help direct risk-assessment and monitoring of patients undergoing treatment of IMI.

## 1. Introduction

Invasive mold infections (IMI) are leading causes of morbidity and mortality in patients with hematological malignancies undergoing intensive chemotherapy or hematopoietic cell transplantation (HCT) [1,2]. Principally, invasive aspergillosis (IA) in patients with hematological malignancies is associated with significant morbidity and mortality and may cause critical delays in time-sensitive treatment regimens [3,4]. The 12-week mortality rate associated with IA has remained around 30% in the 20 years since the introduction of voriconazole as the standard of care [5,6,7]. Review and classification of post-IA mortality suggests that most deaths directly attributable to IA occur within six weeks of diagnosis [8].

Frequently-reported risk factors for death among patients with IMI include the severity and duration of neutropenia [4,9], type and duration of cytotoxic therapy [1,8,10], treatment with amphotericin B (versus voriconazole) [8], receipt of allogeneic HCT, and graft-versus-host disease (GVHD) [4]. However, the relative contribution of host, disease, and treatment-related factors to mortality as a function of time following diagnosis of IMI is unclear [3,4].

Here, we reviewed patients diagnosed with IMI at a tertiary care medical center to define the predominant risk factors during different time periods after the diagnosis.

## 2. Materials and Methods

### 2.1. Study Design

This was an observational, retrospective cohort study of patients with hematological malignancies diagnosed with IMI at the Tel Aviv Sourasky Medical Center (TASMC), a tertiary level, 1500-bed academic hospital, between the years 2007–2016. Inclusion criteria were diagnosis of hematological malignancy; possible, probable, or proven IMI according to the EORTC/MSG criteria [11]; and available health data at least 12 weeks following IMI diagnosis or up to the time of death if earlier than 12 weeks after diagnosis. Clinical data were retrieved from patient medical records, and causes of death for up to 1 year after IMI diagnosis were reviewed and classified by 2 authors (EY and RB).

This study was approved by the TASMC ethics committee (No. TLV 0046-16). Requirement of informed consent was waived due to the retrospective observational nature of the study.

### 2.2. Clinical Management

Recipients of HCT and patients with neutropenia following remission-induction therapy for acute leukemia were accommodated in positive-pressure, HEPA-filtered, single-occupant rooms. Patients were screened twice weekly with serum galactomannan testing. Patients presenting with fever lasting over 96 h or accompanied by respiratory signs and symptoms underwent high-resolution computerized tomography (CT) of the chest, paranasal sinuses, and abdomen. If findings consistent with IMI were found, empirical mold-active therapy was initiated, and bronchoscopy with bronchoalveolar lavage (BAL) was performed, usually within 24 h.

### 2.3. Microbiological Methods

BAL fluid specimens were examined using direct microscopy and Gram staining. Culture was performed on Sabouraud’s dextrose agar at 30 °C and 35 °C. Fungal identification was performed using PCR and sequencing of the 16S–23S rRNA intergenic spacer region. The galactomannan index was determined in serum and BAL fluid using the Platelia EIA Aspergillus Ag assay (Bio Rad, Hercules, CA, USA).

### 2.4. Data Collection

Patient demographic and clinical information were entered in a structured computerized database. The Charlson score was used to summarize comorbidity data [12]. Type of malignancy, cytogenetics, status of malignancy (remission, relapse, or refractory) at the time of IMI diagnosis, and all related cytotoxic and immunomodulating therapeutics were recorded. Neutropenia was defined as absolute neutrophil count (ANC) < 500 cells/microliter, severe neutropenia as ANC < 100 cells/microliter, and prolonged neutropenia as neutropenia lasting >10 days. Neutropenia at the time of IMI diagnosis, total number of days with neutropenia within 30 days before diagnosis, and duration of neutropenia after diagnosis were recorded.

IMI-related data included results of fiberoptic nasal and sinus examination, chest and sinus CT, culture results, histopathology and galactomannan index in serum and BAL fluid. Response to therapy was assessed at 3 weeks and 6 weeks after diagnosis of IMI using MSG/EORTC consensus criteria and categorized as complete response, partial response, stable response, or progression of disease [13].

Deaths up to 12 weeks after diagnosis were analyzed and classified by cause of death as IMI related, infectious non-IMI related, and non-infectious. Cause of death as documented in medical records was taken into consideration but could be overruled by the authors. Death was classified as IMI related if IMI was judged to be unresolved at the time of death, and no alternate cause of death was noted by the treating physicians or apparent from the medical record review. Non-IMI related death was determined when IMI was judged to be stable or resolved, and other likely causes of death were identified [13]. The final classification of death was agreed upon for each patient by both reviewers.

### 2.5. Statistical Analyses

Patient, disease, and treatment variables were described within each patient cohort and defined according to survival status using number (percentage) for categorical variables and value (interquartile range) for continuous variables.

Kaplan–Meier analyses and the log-rank test were performed to determine the effect of each covariate on survival curves. Variables found to be significantly associated with all-cause mortality (*p* < 0.05) were further assessed using Cox proportional hazards modeling. Response to treatment at 3 or 6 weeks after diagnosis was entered into the Cox regression model as a time-dependent variable (not present at baseline). The proportional hazards assumption was checked for each model by testing the time-dependence of covariates and by assessing Schoenfeld residuals after model fitting.

A type I error of <0.05 was considered statistically significant. Calculations were done in Stata 15.0 (Statacorp, College Station, TX, USA).

## 3. Results

Eighty-six patients with IMI met the study criteria (26 proven, 37 probable, and 23 possible IMI). Acute myeloid leukemia (AML) was the most frequent malignancy (*n* = 38, 44%), followed by non-Hodgkin’s lymphoma (NHL) (*n* = 20, 23%) and acute lymphoblastic leukemia (*n* = 13, 15%). Most patients (92%) had uncontrolled malignancy at the time of IMI diagnosis: 52 (60%) had relapsed or refractory malignancy, and 27 (31%) were undergoing initial remission-induction therapy. Thirty-eight patients (44%) had received HCT prior to onset of IMI (26 allogeneic, 7 autologous, and 5 both allogeneic and autologous transplants). The rate of IMI post HCT was 3.8% (12/308) for autologous HCT and 12.5% (31/247) for allogeneic HCT. Most patients (83%) were neutropenic prior to onset of IMI (Table 1).

Sixty-three patients had a microbiologic diagnosis, including all patients with proven disease; of these, 53 patients (61%) had invasive aspergillosis (16 with proven disease), 7 (8%) had mucormycosis, 2 (2%) fusariosis, and 1 (1%) phaeohyphomycosis (all 10 patients with proven disease). Sites of infection were pulmonary (*n* = 72, 84%), paranasal sinuses (*n* = 14, 16%), soft tissue (*n* = 5, 6%), and central nervous system (*n* = 2, 2%) (Table 1). Serum galactomannan at the time of diagnosis was greater than 0.5 for 25 patients (29%) and greater than 1.5 for 10 patients (12%). Galactomannan was determined in bronchoalveolar lavage fluid for 35 patients, and was positive (index >1.0) in 23 patients (65%) (Table 1).

### 3.1. Survival Analyses

Twenty-nine patients (34%) died within six weeks of IMI diagnosis and 41 (47%) died within 12 weeks of diagnosis. There was no significant variation in the 12-week survival rate over 10 years of study data (*p* = 0.6). Infectious diseases were the leading causes of death overall (39/41, 95%); death was classified as IMI related in 22 (53.6%), infective non-IMI related in 17 (41.4%), and non-infective in 2 (4.8%).

Variables associated with overall 12-week mortality were an underlying diagnosis of lymphoma, autologous HCT, positive serum galactomannan (>0.5) at baseline, treatment with an amphotericin B-based regimen, and IMI progression three and six weeks after diagnosis (Table 1). The mortality rate was not significantly different for patients with possible versus probable or proven IMI (39.1% versus 50.7%, respectively; *p* = 0.46).

The association of lymphoma with survival was dependent on autologous HCT status: 12-week mortality among patients with lymphoma was 100% (8/8) for autologous-HCT recipients versus 50% (8/16) for non-transplanted patients.

A Cox proportional hazards model was fitted using variables that interacted significantly with mortality on bivariate analyses (Table 2). Lymphoma and autologous HCT were entered into the model as interacting variables, and response to treatment was entered as a time-dependent variable. The final model included baseline serum galactomannan index >0.5 (hazard ratio (HR) 2.9, *p* = 0.012), initial treatment with an amphotericin B-based regimen (HR 2.07, *p* = 0.048), treatment-refractory malignancy (HR 2.7, *p* = 0.036), lymphoma, and autologous HCT. Lymphoma and autologous HCT were not retained in the model individually, but the presence of both was associated with a hazard ratio of 4.06 (*p* = 0.006).

### 3.2. Early and Late Mortality

To define the risk period for disease-related mortality, we analyzed the time distribution of IMI-associated and non-associated deaths after IMI diagnosis. Of 41 deaths that occurred within 12 weeks of diagnosis, 21 (51.2%) were designated as IMI related, 16 (39.0%) were associated with non-IMI infections, and 4 (9.7%) were non-infection related. IMI related deaths occurred from 2 to 45 days after IMI diagnosis (median, 13 days; IQR, 8 to 39 days). Of 30 deaths that occurred within 45 days of diagnosis, 21 (70.0%) were attributed to IMI, 8 (26.6%) were attributed to bacterial sepsis, and 1 (3.3%) was non-infection related. In contrast, the 11 deaths that occurred from 45 to 84 days after diagnosis were attributed to bacterial sepsis (8 patients, 72.7%) and non-infective causes (3 patients, 27.2%). There were no IMI-related deaths >45 days after diagnosis. We therefore defined early (0 to 45 days) and late (46 days or later) post-IMI periods and assessed the association of host, infection, and treatment related variables with mortality within each period.

Death in the early post-IMI period (<45 days) was associated with a diagnosis of lymphoma (HR 2.05, 95% CI 0.98–4.26), autologous HCT (HR 3.74, 95% CI 1.70–8.20), serum galactomannan index >0.5 (HR 2.37, 95% CI 1.09–5.13), treatment with amphotericin B (HR 2.71, 95% CI 1.23–5.94), and disease progression at three weeks (HR 54.04, 95% CI 7.33–398.35). The hazard ratio for patients with full, partial, or stable responses at three weeks was 0.018 (95% CI 0.002–0.13; *p* < 0.0001). The Cox regression model for early mortality included lymphoma and autologous HCT (interacting host variables), amphotericin B-based therapy, serum galactomannan index >0.5, and IMI progression at three weeks (Table 2).

Death in the late post-IMI period (>45 days) was associated with relapsed or refractory malignancy (HR 7.62, 95% CI 0.97–59.60), progression of IMI at three weeks (HR 3.66, 95% CI 1.11–12.03), and a baseline galactomannan index >1.5 (HR 7.05, 95% CI 1.34–36.95). The Cox regression model for late mortality included refractory malignancy and serum galactomannan index >1.5 (Table 2).

## 4. Discussion

In this observational cohort study, 47% of patients with hematological malignancy and IMI died within 12 weeks, and all cases of IMI-attributable deaths occurred within 45 days of diagnosis. Risk factors for death differed between patients who died within 45 days of IMI diagnosis and those who died later. Specifically, early mortality was associated with IMI-related factors (positive serum galactomannan, disease progression three weeks after starting treatment), treatment factors (amphotericin B), and patient factors (lymphoma and autologous HCT). Risk factors for late (>45 days) mortality were refractory hematological malignancy and a high baseline serum galactomannan index (>1.5).

Our findings are consistent with previous studies showing temporal variation in IA-attributable mortality. Wingard et al. [8]. reviewed causes of death using data from a prospective randomized controlled trial comparing voriconazole and amphotericin B for the treatment of IA [5]. They found that 68% of deaths within six weeks of diagnosis were attributable to IA, whereas only 24% of deaths that occurred during weeks 7 to 12 were IA-attributable [8]. Garcia-Vidal et al. [14] reviewed retrospective data on patients with IA at three hospitals in Barcelona, Spain. Similar to our findings, almost all IA-related deaths occurred within 45 days of diagnosis. The risk of IA-related death was increased in patients with chronic liver disease and decreased in patients treated with voriconazole [14].

Unlike these studies, we analyzed retrospective observational data from a single center spanning a decade. We included all IMIs, recognizing that in clinical practice, many mold infections in the hematological population are not characterized microbiologically. Our analysis of risk factors was not limited to deaths that were designated as directly attributable to IMI. In addition, we incorporated consensus criteria for assessing response to treatment into our analyses [13].

The mortality rate of patients with hematological malignancies and IMI has improved following the introduction of voriconazole [5,15], but it remains high (around 50%) three months after the onset of infection [15]. Whether this mortality rate reflects the limited efficacy of available treatments against pathogenic molds or other associated host and disease factors is unclear. Better understanding of the factors contributing to patient mortality is needed to prioritize research and target diagnostic and clinical monitoring efforts.

Detection of galactomannan in serum may allow early initiation of antifungal treatment [16]. Therefore, the association of elevated galactomannan with mortality might seem paradoxical. However, other groups [17,18] have shown that the magnitude of the galactomannan index in the serum—but not BAL fluid—of HSCT recipients is an independent predictor of mortality. Fisher et al. hypothesized that the galactomannan index correlates with overall fungal burden and that higher index is associated with more angioinvasion at the pulmonary alveoli [17]. Moreover, the galactomannan index may be associated with subclinical hematogenous dissemination of *Aspergillus* species, not detected by conventional imaging modalities. Of note, while detectable serum galactomannan was associated with early mortality in our study, a galactomannan index above 1.5 was also predictive of late mortality, which was not directly attributable to IMI. This finding suggests that the galactomannan index may be an indirect marker of overall patient health and immune competence, which are in turn linked to bacterial sepsis and death.

The association between lymphoma, autologous transplantation, and mortality was an unexpected finding of this study. Most of these patients had chemo-refractory lymphoma and were transplanted in the earlier years of this study, when immunotherapy was not yet available. Thus, these patients were heavily pretreated prior to autologous HCT and usually experienced prolonged durations of aplasia. Currently, such patients are treated early with either CAR-T cell infusion or other specific antibodies, resulting in a lower risk for IMI following autologous HCT. Nevertheless, we postulate that the risk of IMI may be underappreciated in heavily immunosuppressed patients with non-Hodgkin’s lymphoma, resulting in delays in diagnosis, increased time to initiation of appropriate antifungal treatment, and increased risk of adverse outcomes of IMI.

Response to treatment, assessed three weeks after initiation of treatment using repeated CT imaging of the chest and paranasal sinuses and changes in the baseline galactomannan index, was strongly predictive of early and overall mortality. A landmark study by Caillot et al. [19] showed that the volume of pulmonary lesions of invasive aspergillosis increases during the first week of therapy and stabilizes during the second week. Thus, three weeks after initiating treatment may be an optimal time point to assess early radiographic response. Of note, Vehreschild et al., using meticulous serial CT analysis, found that the trend of lesion volume between day 7 and 14 after starting treatment correlated with patient survival [20]. Similarly, previous studies have shown that increasing galactomannan index values portend a poor outcome for patients with invasive aspergillosis [21,22] and may assist in differentiating immune-reconstitution inflammatory syndrome from progressive aspergillosis [23]. Finally, initial treatment with amphotericin B deoxycholate, which was used as empirical treatment of patients with persistent febrile neutropenia up until 2010, was associated with lower early and overall survival of patients with IMI. This finding is consistent with the results of a randomized controlled trial showing the inferiority of this drug as compared with voriconazole for the treatment of invasive aspergillosis [5].

Limitations of this study are inherent to its observational, retrospective design. Being a single-center study, findings may not be generalizable to other settings. Additional limitations are the size of the patient cohort and the potential heterogeneity in clinical practice during the 10 year study period.

In conclusion, in this observational, single-center cohort of patients with hematological malignancies and invasive mold infections, death related to fungal disease occurred within 45 days of diagnosis, and different patient, treatment, and disease covariates were associated with early versus late mortality. These findings have implications for the use of diagnostics for patient monitoring and follow-up but require validation in other settings.

## Figures and Tables

**Table 1:** Characteristics of patients according to 12-week survival status.

Variable	Outcome at 12 Weeks		Log-Rank *p* Value	Total *n* = 86
	Survival *n* = 45	Death *n* = 41		
Male Sex	30 (66.6)	26 (63.4)	0.84	56 (65.1)
Age, years, median (IQR)	52 (36–66)	51 (37–63)	0.77	52 (36–66)
Healthcare tourism	9 (20.0)	8 (19.5)	0.76	17 (19.8)
Charlson index, median (IQR)	3 (2–3)	3 (2–3)	0.52	6 (7)
Hematological Malignancy				
AML	24 (53.3)	14 (34.1)	0.091	38 (44.1)
ALL	8 (17.7)	5 (12.2)	0.39	13 (15.1)
CLL	6 (13.3)	4 (9.7)	0.53	10 (11.6)
NHL	7 (15.5)	13 (31.7)	0.091	20 (23.2)
Hodgkin’s lymphoma	1 (2.2)	3 (7.3)	0.031	4 (4.6)
CML	1 (2.2)	3 (7.3)	0.30	4 (4.6)
Myeloma	1 (2.2)	1 (2.4)	0.79	2 (2.3)
MDS	2 (4.4)	3 (7.3)	0.58	5 (5.8)
Hematopoietic stem cell transplant (HSCT)	16 (35.5)	22 (53.6)	0.12	38 (44.1)
Allogeneic HSCT	15 (33.3)	16 (39.0)	0.35	31 (36.0)
Autologous HSCT	2 (4.4)	10 (24.3)	0.0006	12 (13.9)
Conditioning				
Ablative	15 (93.7)	19 (86.3)	0.36	34 (89.4)
Non-ablative	1 (6.2)	3 (13.6)	0.41	4 (10.5)
ATG	8 (17.7)	12 (29.2)	0.094	20 (23.2)
Status of malignancy				
Relapse/Refractory	22 (48.8)	30 (73.1)	0.028	52 (60.4)
Remission induction	19 (42.2)	8 (19.5)	0.036	27 (31.4)
Remission	4 (8.8)	3 (7.3)	1.0	7 (8.1)
Host Factors				
Neutropenia (ANC < 500/mcL)	40 (88.8)	32 (78.0)	0.12	72 (83.7)
Severe neutropenia (ANC < 100/mcL)	25 (55.5)	25 (60.9)	0.64	50 (58.1)
Neutropenia duration (days)	24 (14–49)	23 (12–32)	0.22	23.5 (14–40)
GVHD	13 (28.8)	7 (17.0)	0.24	20 (23)
Corticosteroids	11 (24.4)	13 (31.7)	0.52	24 (27.9)
Calcineurin inhibitor	19 (42.2)	13 (31.7)	0.28	32 (37.2)
EORTC/MSG classification				
Possible	14 (31.1)	9 (21.9)	0.16	23 (26.7)
Probable	18 (40)	19(46.3)	0.08	37 (43.0)
Proven	13 (28.8)	13 (31.7)	0.83	26 (30.2)
Fungal pathogen				
Aspergillus	25 (55.5)	28 (68.2)	0.26	53 (61.6)
Mucorales	4 (8.8)	3 (7.3)	0.59	7 (8.1)
Fusarium	2 (4.4)	0 (0)	0.24	2 (2.3)
Site of Infection				
Pulmonary	36 (80.0)	36 (87.8)	0.23	72 (83.7)
Sinus	6 (13.3)	8 (19.5)	0.38	14 (16.2)
Soft tissue	2 (4.4)	3 (7.3)	0.58	5 (5.8)
CNS	1 (2.2)	1 (2.4)	0.79	2 (2.3)
Chest CT				
Angioinvasive pattern	18 (40.0)	15 (36.5)	0.83	33 (38.3)
Airway invasive pattern	3 (6.6)	2 (4.8)	0.69	5 (5.8)
Combined pattern	12 (26.6)	14 (34.1)	0.38	26 (30.2)
Halo sign	8 (17.7)	7 (17.0)	0.97	15 (17.4)
Serum GMI > 0.5	8 (20.0)	17 (51.5)	0.0033	25 (29)
Maximal serum GMI, median (IQR)	0.32 (0.2–0.46)	0.6 (0.3–1.6)	0.008	0.36 (0.28–0.7)
BALF GMI > 1.0	11 (55.0)	12 (80.0)	0.18	23 (65.7)
Initial antifungal treatment				
Voriconazole	25 (55.5)	23 (56.1)	0.90	48 (56)
Posaconazole	3 (6.6)	1 (2.4)	0.33	4 (4.6)
Amphotericin B-based ^a^	4 (8.8)	11 (26.8)	0.006	15 (12.8)
Echinocandin	1 (2.2)	1 (2.4)	0.95	2 (2.3)
Non-active drug	8 (17.7)	4 (9.7)	0.30	12 (13.9)
Response to treatment, 3 weeks				
Full	4 (8.8)	1 (2.4)	0.25	5 (5.8)
Partial	34 (75.5)	5 (12.2)	<0.001	39 (45.3)
Stable	0 (0)	1 (2.4)	0.65	1 (1.1)
Progression	7 (15.5)	34 (82.9)	<0.001	41 (47.7)
Response to treatment, 6 weeks				
Full	12 (26.6)	1 (2.4)	0.022	13 (15.1)
Partial	22 (48.8)	3 (7.3)	0.001	25 (29.0)
Stable	2 (4.4)	0 (0)	1.0	2 (2.3)
Progression	6 (13.3)	11 (26.8)	0.25	17 (19.7)

All values represent number of patients (percent within survival group), unless specified otherwise. AML, acute myeloid leukemia; NHL, non-Hodgkin lymphoma; ALL, acute lymphocytic leukemia; CLL, chronic lymphocytic leukemia; HD, Hodgkin’s disease; CML, chronic myeloid leukemia; MM, multiple myeloma; MDS, myelodysplastic syndrome; HSCT, hematopoietic stem cell transplant; ATG, anti-thymocyte globulin; GVHD, graft-versus-host disease; IA, invasive aspergillosis; BALF, bronchoalveolar lavage fluid; GMI, galactomannan index; ANC, absolute neutrophil count. ^a^ Amphotericin B deoxycholate (*n* = 14), liposomal amphotericin B (*n* = 1).

**Table 2:** Cox regression models for early, late, and overall mortality.

Variable	Early Mortality	Late Mortality	Overall Mortality
Lymphoma/Autologous HCT	4.51 (1.67–12.17)		4.06 (1.5–10.9)
Amphotericin B treatment	2.33 (0.81–6.64)		2.07 (0.76–5.60)
Serum GMI > 0.5	2.80 (1.03–7.64)		2.9 (1.2–6.9)
Serum GMI > 1.5		11.08 (1.8–67.5)	
Progression at 3 weeks	36.88 (4.79–283.87)		706.2 (6.6–75,179.6)
Treatment-refractory malignancy		8.48 (1.40–51.3)	2.7 (1.06–6.8)

All values represent hazard ratio (95% confidence intervals).

## Data Availability

Supporting data is available from the corresponding author upon request.
